# Effect of Laser Selective Melting on the Microstructure and Properties of Martensitic Stainless Steel After Annealing Treatment

**DOI:** 10.3390/ma18020354

**Published:** 2025-01-14

**Authors:** Biao Zhou, Shuai Huang, Tianyuan Wang, Zijun Zhao, Bingqing Chen

**Affiliations:** 3D Printing Research and Engineering Technology Center, Beijing Institute of Aeronautical Materials, Beijing 100095, China; zhoubiao0317@163.com (B.Z.); 15698775910@163.com (T.W.); zzj_9501@163.com (Z.Z.); hwtkjcbq1984@163.com (B.C.)

**Keywords:** stainless steel, selective laser melting, tempering temperature, microstructure, mechanical properties

## Abstract

This work investigated the _0_Cr_16_Ni_5_Mo_1_ stainless steel using laser selective melting (SLM) technology and explored the effect of the tempering temperature on the microstructure and properties. After the tempering treatment, the quenched martensite transformed from a metastable to steady state, and residual austenite was formed. The results indicated that the elongation of the transverse specimen showed an upward trend as the tempering temperature increased, while the elongation of the longitudinal specimen first increased and then decreased. The fracture mode was ductile. There was an obvious fiber, radial, and shear lip zone on the fracture surface of transverse specimens. When the tempering temperature was 650 °C, the shear lip area of the fracture surface was the largest. For longitudinal specimens, there was no obvious zoning on the fracture surface.

## 1. Introduction

Additive manufacturing is a new type of manufacturing technology that is based on a model with parameter settings and aims to obtain actual parts [1,2,3,4]. The combination of additive manufacturing and computer technology has improved the automation forming level. At the same time, the applicability of additive manufacturing materials has greatly developed. At present, the main heat sources for metal additive manufacturing are supplied by laser and electron beam, which can be specifically divided into laser and electron beam additive manufacturing technology. The thermal history, cooling, and solidification of metal additive manufacturing are greatly different from traditional forging, casting, and extrusion forming, resulting in certain differences in the microstructure and properties of parts. Metal additive manufacturing equipment, raw materials, and processes are important factors that affect the final formed parts [5]. Based on the characteristics of different metal additive manufacturing technologies, appropriate forming processes should be selected for different standards and requirements to improve material utilization and promote the development of short-process, integrated, and continuous metal additive manufacturing technology.

SLM technology combines the characteristics of metal additive manufacturing with its unique advantages [6,7,8,9]: (1) It reduces the processing time of the final parts. After the forming process, the part is ready without the need for subsequent machining or post-processing. (2) There are few defects in the parts, and the precision is high. The laser spot diameter formed by SLM reaches a micrometer, and the parts are fused well to achieve a high density, with a scale accuracy of 20–50 μm and low surface roughness. Simultaneously, the solidification speed is fast, the structure is fine, and the performance is good. (3) It can achieve the formation of a wide range of materials. In SLM, process parameters are one of the main influencing factors. The main process parameters are the energy density, laser power, scanning speed, scanning spacing, powder coating thickness, and scanning strategy [10,11]. The cooling speed can be controlled through the laser energy input, and the laser energy density determines the density of SLM-manufactured parts. The changes in laser power and scanning speed can also be presented through changes in the energy density. The scanning distance determines the overlap rate between molten pools and has a significant impact on the formability and microstructure. The effective combination of layer thickness, laser power, and scanning speed is one of the most effective ways to improve SLM efficiency [12,13].

Additive manufacturing forming for 316L stainless steel precision complex parts has received great attention. 316L stainless steel has also been applied in additive manufacturing forming in the medical field due to its lower cost compared to titanium or cobalt chromium alloys, better comprehensive mechanical properties, and good corrosion resistance [14,15]. Due to the presence of numerous defects in some samples, their tensile strength and elongation are relatively low. In order to balance production efficiency and excellent properties, this work conducts different tempering temperatures on the SLM _0_Cr_16_Ni_5_Mo_1_ samples, explores the influence of tempering temperatures on properties, and optimizes the tempering treatment of SLM. _0_Cr_16_Ni_5_Mo_1_ martensitic stainless steel is a type of stainless steel developed by reducing C and increasing Ni and Mo based on the traditional martensitic stainless steel composition. With an increase in the tempering temperature, the impact toughness of stainless steel clearly increases, the microstructure still has a certain martensite orientation, and a large number of adjacent martensite flat noodles boundaries are fused. After appropriate quenching and tempering treatment, the structure is mainly composed of tempered martensite and residual austenite. Tempered martensite can ensure the strength, while residual austenite can improve the plasticity and toughness of the material [16].

## 2. Experimental Materials and Methods

The SLM process was adopted and _0_Cr_16_Ni_5_Mo_1_ stainless steel powder with a particle size of 15–53 μm was selected for additive manufacturing experiments. The chemical composition of the powder is shown in Table 1. The experiment used the laser selective melting forming equipment (EOS-M290, St. Georg, Hamburg, Germany), which was equipped with a 400 W fiber laser with a maximum forming size of 250 × 250 × 325 mm^3^, a single-layer powder coating thickness of 20–100 μm, and a maximum scanning speed of 7000 mm/s. High-purity Ar was used as inert protective gas to control the levels of O, H, N, and other elements.

A stainless steel plate was selected as the substrate, with a laser power of 234 W and a laser scanning rate of 828 mm/s. Transverse test blocks with dimensions of 13.5 × 13.5 × 73 mm^3^ and longitudinal test bars with dimensions of Φ 13.5 mm were obtained as mechanical sample blanks. Each result was the average of five samples. The additive manufacturing sample blank was placed into a heat treatment furnace for the 950 °C insulation and 1 h quenching treatment, and was then air cooled to room temperature. Subsequently, the quenched additive manufacturing tests were placed in the furnace and subjected to tempering treatment at 500 °C, 550 °C, 600 °C, and 650 °C for 4 h, and cooled in air to room temperature. These samples were processed into a tensile sample using a wire-cutting machine. Room-temperature tensile testing was performed using an Instron universal tensile testing machine in accordance with GB/T 228.1-2010 [17]. The clamping end along the axial direction of the specimen (length direction) was ground, polished, and etched with FeCl_3_+HCl alcohol solution. Scanning electron microscopy was used to observe the fracture surfaces and metallographic specimens of each sample, and the effect of tempering temperature on the microstructure and properties of the _0_Cr_16_Ni_5_Mo_1_ stainless steel produced by additive manufacturing was analyzed and compared.

## 3. Results and Discussion

### 3.1. Microstructure and Grain Size

**Figure 1:**
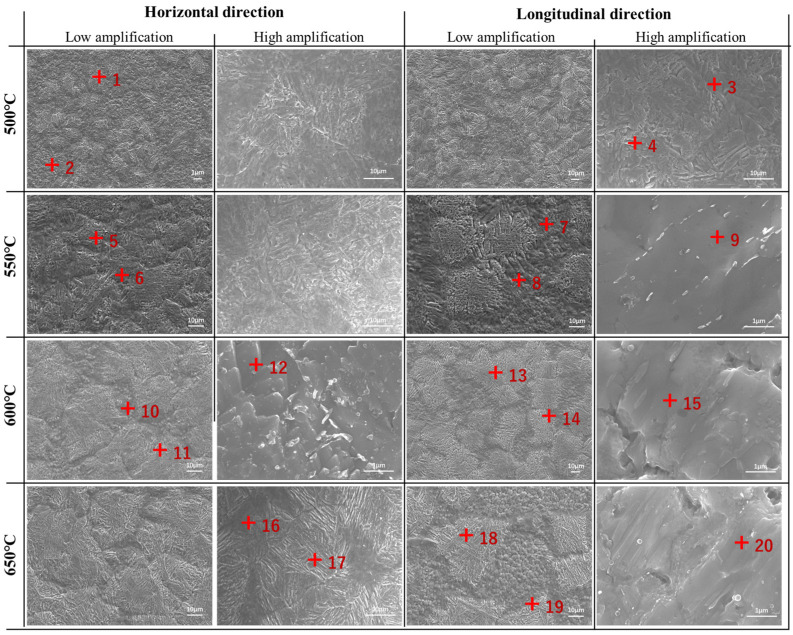
SEM images of microstructure for high- and low-amplification specimens at different tempering temperatures: 500 °C, 550 °C, 600 °C, and 650 °C.

Figure 1 shows the microstructure of transverse and longitudinal specimens at different tempering temperatures, and the chemical composition is shown in Table 2. After tempering, the originally coarse quenched martensite transformed from a metastable to steady state, forming a relatively fine tempered martensite. With the increase in the tempering temperature, the width of martensite in transverse samples increased, and the martensite flat noodles were more clearly visible. After 4 h of holding the sample at a tempering temperature of 500 °C, the quenched martensitic in the transverse specimen became a relatively small steady-state tempered martensitic structure. The higher the tempering temperature, the shorter the time taken for the quenched martensite to transform into steady-state tempered martensite [16,18].

From Figure 1, it is clear that the microstructure in the longitudinal sample still retained its original coarse morphology at tempering temperatures of 500 °C and 550 °C, unlike the transverse sample. Fine martensitic structures had formed on the coarse martensite. When the tempering temperatures were 600 °C and 650 °C, the variation in the longitudinal specimen was similar to that of the transverse specimen. This indicated that at tempering temperatures of 500 °C and 550 °C, the quenched martensite in the longitudinal specimen, which was originally in a metastable state, did not fully transition to a steady-state tempered martensitic structure. When the tempering temperatures were 600 °C and 650 °C, the transformation of the longitudinal specimen to steady-state tempered martensite had been basically completed.

**Figure 2:**
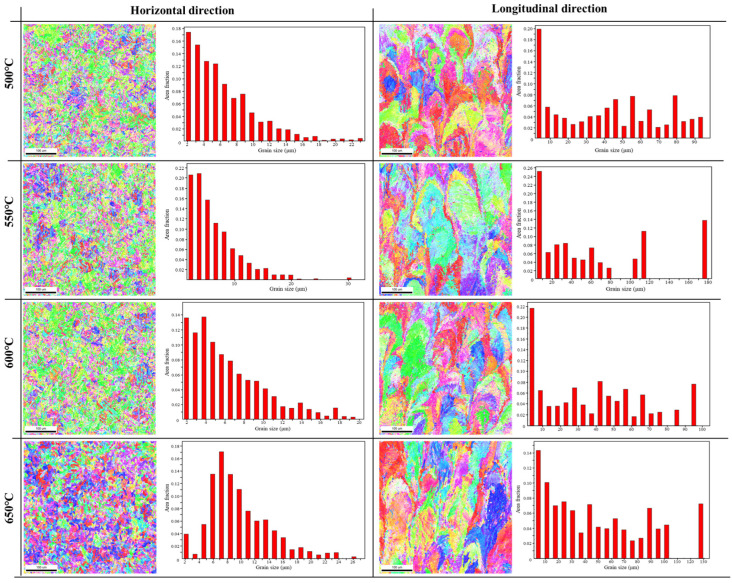
EBSD images of microstructure and grain size for specimens at different tempering temperatures: 500 °C, 550 °C, 600 °C, and 650 °C.

When the tempering temperatures were 500 °C and 550 °C, the residual austenite slightly decreased with the increase in the tempering temperature, as shown in Figure 2. When the tempering temperature was 600 °C, the residual austenite significantly increased and reached its maximum. When the tempering temperature was 650 °C, the residual austenite decreased. For transverse specimens, the higher the tempering temperature, the higher the content of reverse austenite in the specimen. For longitudinal specimens, when the tempering temperatures were 500 °C and 550 °C, the original quenched microstructure in the longitudinal specimen was not completely transformed into a steady-state tempered martensite microstructure. Therefore, higher tempering temperatures would result in more quenched martensite and residual austenite in the original quenched microstructure transforming into steady-state tempered martensite. When the tempering temperature was 600 °C, the microstructure was basically transformed into steady-state tempered martensite. Compared with the samples at tempering temperatures of 500 °C and 550 °C, the steady-state tempered martensite in the samples at this tempering temperature underwent a reverse transformation. Therefore, the residual austenite content increased significantly after cooling. When the tempering temperature was 650 °C, the reverse austenite in the sample continued to increase. However, due to the high tempering temperature, the stability of reverse austenite decreased, and secondary quenched martensite formed after cooling [19,20]. Therefore, the residual austenite in the sample decreased at this tempering temperature. In addition, by comparing the variation in the residual austenite content with the tempering temperature in transverse and longitudinal specimens, it could be found that the stability of reverse austenite in transverse specimens was higher than that in longitudinal specimens.

### 3.2. Effect of Tempering Temperature on Properties of Samples

**Figure 3:**
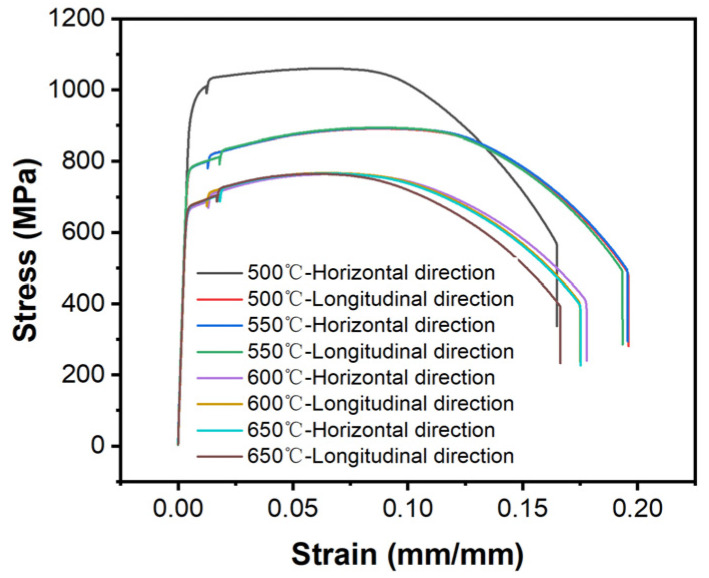
Stress–strain curves at different tempering temperatures and directions.

**Figure 4:**
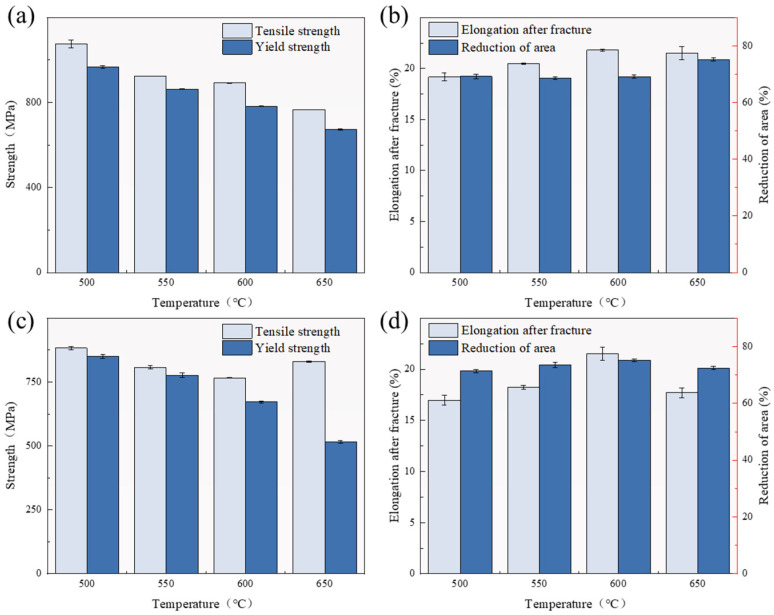
Mechanical properties of samples at different tempering temperatures: (**a**) transverse yield strength and tensile strength; (**b**) transverse elongation and cross-sectional shrinkage rate; (**c**) longitudinal yield strength and tensile strength; (**d**) longitudinal elongation and reduction in area.

Figure 3 shows the stress–strain curves at different tempering temperatures and directions. When the tempering temperature was 500–600 °C, the tensile strength of both the transverse and longitudinal specimens decreased (Figure 4a,c). When the tempering temperature was 650 °C, the tensile strength of the transverse specimen hardly changed, while the tensile strength of the longitudinal specimen increased significantly. For transverse specimens, as the tempering temperature increased, the reduction in dislocation density and residual stress in the specimen, as well as the coarsening of tempered martensite, increased, which resulted in a decrease in the tensile strength. When the tempering temperature was 650 °C, the dislocation density and residual stress were similar to those at a tempering temperature of 600 °C. Compared to the sample at a tempering temperature of 600 °C, its tensile strength changed little. For longitudinal specimens, although the residual austenite content slightly decreased, the increase in the tempering temperature promoted the transformation of original quenched martensite to tempered martensite, reduced residual stress, and the dislocation density decreased with the increase in the tempering temperature. Therefore, the higher the tempering temperature, the lower the tensile strength of the tempered specimen. When the tempering temperature was 600 °C, the residual austenite was the highest, and the residual stress and dislocation density further decreased, resulting in a continued decrease in its tensile strength. When the tempering temperature was 650 °C, the stability of the reverse transformation austenite in the sample decreased during the tempering process, and secondary quenched martensite was formed during the cooling process, resulting in an increase in its tensile strength.

Figure 4b,d show the elongation of transverse and longitudinal additive specimens at different tempering temperatures. The elongation of the transverse tensile specimen increased with the increase in the tempering temperature. The elongation of the longitudinally stretched specimen showed a trend of first increasing and then decreasing with the increase in tempering temperature. When the tempering temperature was 600 °C, its elongation was the highest. For transverse tensile specimens, when the tempering temperature was 500–600 °C, the decrease in dislocation density and residual stress, as well as the residual austenite content, increased with the increase in tempering temperature, resulting in an increase in plasticity and elongation. When the tempering temperature was 650 °C, the dislocation density and residual stress in the sample after tempering were similar to those at a tempering temperature of 600 °C. However, the residual austenite content in the sample continued to increase after tempering, resulting in a further increase in elongation. This also indicated that the change in the residual austenite content had little effect on the tensile strength of transverse specimens, but had a significant impact on their elongation.

For longitudinal specimens, when the tempering temperature was 500 °C and 550 °C, the higher the tempering temperature, the greater the degree of transformation from original quenched martensite to tempered martensite, the lower the residual stress and dislocation density, the better the plasticity, and the higher the elongation; when the tempering temperature was 600 °C, the residual austenite content in the tempered sample reached its maximum, and the residual stress and dislocation density further decreased, resulting in a further increase in its elongation; and when the tempering temperature was 650 °C, the secondary quenched martensite formed in the tempered sample led to a decrease in its plasticity and subsequently a decrease in its elongation. By comparing the mechanical properties of transverse and longitudinal specimens, it can be found that transverse specimens had better mechanical properties compared to longitudinal specimens. This may be due to the presence of more pore defects in the longitudinal specimens. There were two types of pore defects: circular pores formed during the additive manufacturing process due to incomplete gas escape. Another type of pore was formed during the additive manufacturing process, where the gaps in the previous layer were not filled by the subsequent layer of material, resulting in unfused holes, usually thin and flat holes perpendicular to the stacking direction. Hole defects had a significant impact on the mechanical properties, especially the second type of hole. The tip of an unfused hole was prone to stress concentration during tensile testing, which accelerated crack propagation [21,22,23]. Compared to transverse specimens, longitudinal specimens had more stacking layers and were more prone to void defects, resulting in poorer mechanical properties.

**Figure 5:**
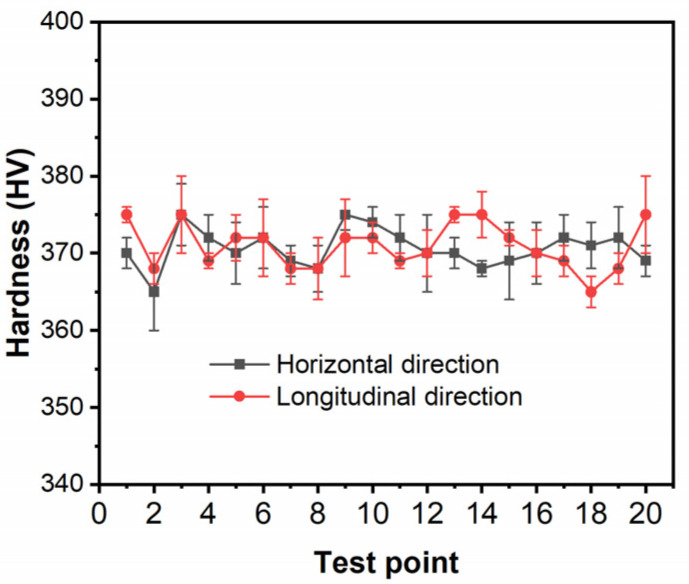
Hardness of the _0_Cr_16_Ni_5_Mo_1_ martensitic stainless steel in different directions.

Figure 5 shows the hardness test results of _0_Cr_16_Ni_5_Mo_1_ martensitic stainless steel in different directions. It can be seen that the hardness of the stainless steel fluctuates slightly around 370 HV, and the change in test direction is not sensitive. This once again proves that the microstructure of _0_Cr_16_Ni_5_Mo_1_ martensitic stainless steel is not greatly affected by direction.

### 3.3. Fracture Surface of Specimens at Different Tempering Temperatures

**Figure 6:**
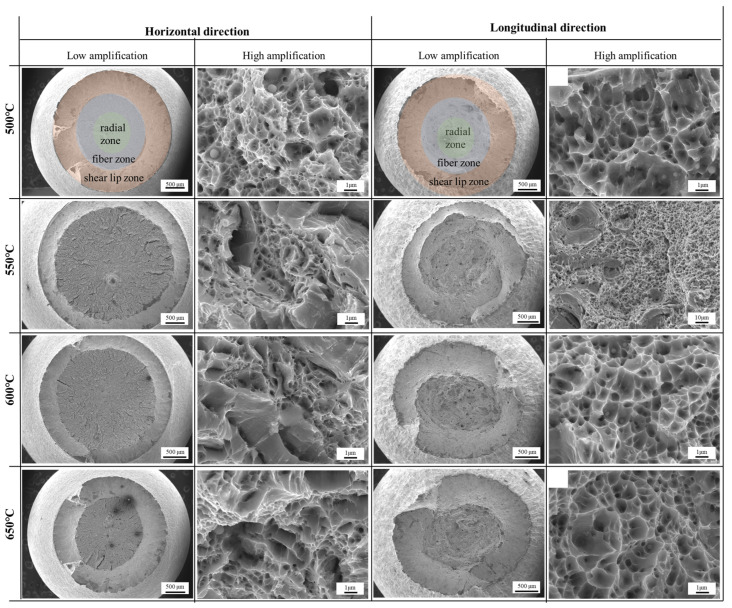
High- and low-amplification transverse and longitudinal fracture morphology of samples at different tempering temperatures: 500 °C, 550 °C, 600 °C, and 650 °C.

Figure 6 shows the fracture morphology of transverse and longitudinal specimens at different tempering temperatures. Based on the morphology fracture, the transverse specimen fracture at different tempering temperatures had three distinct regions: the fiber zone, radial zone, and shear lip zone. Among them, pore defects were found in the fiber zone. The radial area of the transverse specimen fracture surface was the largest when the tempering temperature was between 500 and 600 °C. However, when the tempering temperature was 650 °C, the radial area of the transverse specimen fracture surface decreased significantly, while the shear lip area increased significantly. This also indicated that the plasticity of the transverse specimen was the best at this tempering temperature. The fracture surfaces were all ductile dimples with clearly visible tearing edges, and the fracture mode was ductile fracture. When the tempering temperature was between 500–600 °C, as the tempering temperature increased, the tear edges in the radial zone of the transverse specimen fracture became higher, and the toughness dimples merged into “radial edge lines”, with a deeper depth [24]. This indicated that the crack tip was passivated, the energy required for propagation increased, the tear work increased, and the plasticity was enhanced. When the tempering temperature was 650 °C, the number of “radial ridges” in the transverse specimen fracture surface decreased, the depth became shallower, and the number of toughness dimples increased. This means that at this tempering temperature, the crack propagation rate in the radial zone increased. There is no clear zoning on the fracture surface of the longitudinal specimens at different tempering temperatures. In addition, compared to the transverse specimen, there are more pore defects in the fracture surface of the longitudinal specimen, which is the reason for the inferior mechanical properties of the longitudinal specimen. Similar to the transverse specimen, the fracture mode of the longitudinal specimen was ductile fracture. When the tempering temperature was 500–600 °C, as the tempering temperature increased, the toughness dimples in the fracture surface of the sample showed a trend of increasing and deepening, and the plasticity was enhanced. When the tempering temperature was 650 °C, the depth of ductile dimples in the fracture surface of the sample became shallower and the plasticity deteriorated.

## 4. Conclusions

1. With the increase in tempering temperature, the width of tempered martensite in the transverse sample became larger, and the martensite flat noodles were more clearly visible. The residual austenite content showed a trend of first decreasing, then increasing, and finally decreasing again.
2. The tensile strength of both transverse and longitudinal specimens decreased with the increase in tempering temperature. When the tempering temperature was 650 °C, the tensile strength of the transverse specimen did not change significantly, while the tensile strength of the longitudinal specimen increased.
3. As the tempering temperature increased, the elongation of the transverse specimen showed an increasing trend, while the elongation of the longitudinal specimen first increasing and then decreasing. There were obvious fiber zones, radial zones, and shear lip zones on the fracture surface, and the fracture mode of both horizontal and longitudinal specimens was ductile fracture.

## Figures and Tables

**Table 1:** Chemical composition of _0_Cr_16_Ni_5_Mo_1_ stainless steel powder (wt. %).

C	Cr	Ni	Si	Mn	Mo	P	S	Fe
0.0053	16.34	4.90	0.40	0.41	0.79	0.012	0.0068	Bal.

**Table 2:** The chemical composition corresponding to the microstructure in Figure 1 (at.%).

Point	Si	Mo	Cr	Mn	Fe	Ni
1	0.36	0.2	18.12	0.54	76.13	4.66
2	0.32	0.52	18.84	0.66	75.43	4.22
3	0.35	0.38	18	0.79	75.95	4.53
4	0.37	0.38	18.11	0.82	75.52	4.6
5	0.33	0.36	18.18	0.88	75.49	4.75
6	0.55	0.53	18.45	0.78	75.26	4.42
7	0.53	0.67	18.04	0.9	75.5	4.35
8	0.43	0.6	17.91	1.18	75.27	4.61
9	1.01	0.97	17.51	0.85	74.78	4.88
10	0.75	0.63	18.47	0.92	74.47	4.76
11	0.69	0.55	18.09	0.62	75.76	4.29
12	0.53	0.45	18.34	0.69	75.71	4.29
13	0.65	0.43	18.13	1.05	74.85	4.9
14	1.06	0.87	18.8	0.78	74.18	4.21
15	0.36	0.37	18.33	0.73	75.71	4.49
16	0.62	0.7	18.33	0.65	75.05	4.65
17	0.7	0.55	18.45	0.77	74.76	4.77
18	0.8	0.7	18.03	0.46	75.35	4.65
19	0.74	0.52	18.26	0.83	75.06	4.59
20	0.73	0.61	18.11	0.48	75.91	4.17

## Data Availability

The original contributions presented in this study are included in the article. Further inquiries can be directed to the corresponding author(s).
